# Asymmetry in the evolution of the male and female germlines

**DOI:** 10.1371/journal.pbio.3003907

**Published:** 2026-07-24

**Authors:** Carina F. Mugal, Marie Sémon

**Affiliations:** 1 Laboratory of Biometry and Evolutionary Biology, CNRS, UMR 5558, University of Lyon 1, Villeurbanne, France; 2 Laboratory of Biology and Modeling of the Cell, Ecole Normale Supérieure de Lyon, CNRS, UMR 5239, Inserm, U1293, Université Claude Bernard Lyon 1, Lyon, France

## Abstract

Reproductive organs are under strong selective pressure during evolution. This primer discusses a recent PLOS Biology manuscript that has revealed asymmetric evolutionary divergence in Drosophila reproductive organs, wherein meiotic cell types in the testis emerge as hotspots of rapid change and ovary cell types remain more conserved.

## Teaser

A new study in *PLOS Biology* shows that in *Drosophila,* patterns of molecular evolution differ between genes expressed during oogenesis and spermatogenesis. Their evolutionary framework provides a basis for better understanding the link between molecular evolution of reproductive organs and the speciation process.

The transition from classic bulk-tissue transcriptomics to single-cell or single-nucleus transcriptomics has paved the way to dissect the cellular complexity of tissues and organs and study gene regulation at its basal resolution of individual cell types. Notably, this technological advance has proven particularly powerful for reconstructing the dynamic of cell differentiation and organ development, where its application to gonads has emerged as a relevant means to study gametogenesis [1]. Gametogenesis, the process of forming mature germ cells, involves the interplay of many differentiating cell types. Despite its complexity, cross-species comparisons at the bulk level have revealed the testis to be a particularly rapidly evolving organ at the molecular level in both mammals [2] and insects [3], matching morphological variations in testis sizes, sperm production rates, and sperm morphologies. Although comparative studies on ovaries remain rare, they do not mirror this trend [2,3].

While comparative transcriptomics at the bulk-level mask possible peculiarities across different stages of gametogenesis, pioneering applications of single-cell transcriptomics in an evolutionary biology context remain largely based on the comparison of gonads between distantly related species, with a focus on spermatogenesis, and little insight on the evolutionary patterns of oogenesis [4]. To complement these earlier findings, a recent study published in *PLOS Biology* by Hariyani and colleagues [5] now documents spermatogenesis and oogenesis in closely related *Drosophila* species at the single-nucleus level. Specifically, an important novelty of this work is the investigation of rates of molecular evolution from the intra-specific to the inter-specific scale, in both female and male gonads, which provides a solid foundation for evolutionary interpretations.

**Fig 1:**
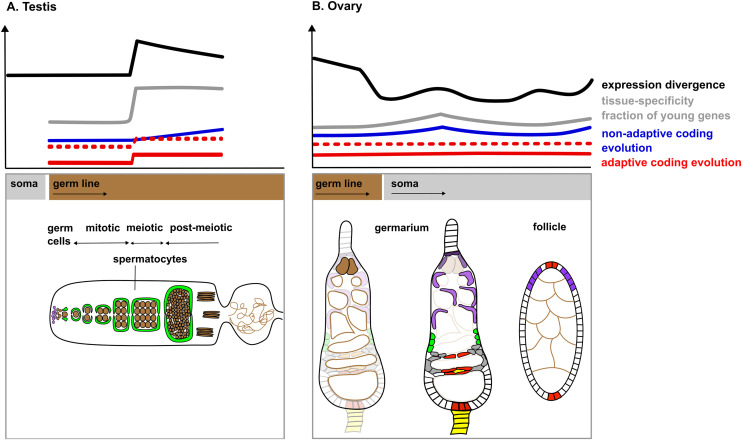
Patterns of molecular evolution for genes expressed during spermatogenesis and oogenesis. **(A)** Testis cell types differ in terms of molecular evolutionary rates, with stronger divergence in gene expression and their coding sequences in spermatocytes. **(B)** Ovary cell types are more homogeneous in terms of divergence in expression and sequences. Both panels, line graphs: Bold lines correspond to observations for genes on the autosomes, the red dashed line corresponds to X-linked genes. The size of the bars below the line graphs represents the relative proportions of soma (dark gray) and germ (brown) in the *Drosophila* gonad. Bottom sketches represent a testis with spermatogenesis, and a germinarium and a follicle for oogenesis (Anterior is up for germaria and follicle). Germ cells are depicted in brown and somatic cells in other colors.

The authors obtain single-nucleus atlases for two separate strains from *Drosophila melanogaster* and one *Drosophila simulans* strain for both male and female gonads. Consistent with earlier findings in terms of cell type composition on similar data [6,7], germ cells represent roughly 80% of all cells recovered from the testes, whereas the fraction of germ cells is only about 20% of all cells recovered from ovaries. Intra- and inter-specific pair-wise comparisons of the transcriptome show strong preservation of cell composition among strains. However, differential gene expression analysis, enabled by the comparison of closely related species, reveals characteristic patterns of gene expression divergence. In the testis, expression divergence peaks in late and maturing primary spermatocytes, while in the ovary it is elevated in early germarium stages and late-stage main body follicle cells (Fig 1). Importantly, divergence in the ovarian transcriptome is significant, although expression divergence is overall stronger in male than female gametogenesis.

In addition to expression divergence, the authors computed a series of metrics classically used in evolutionary analyses to study the evolution of genes expressed at different stages of gametogenesis. Specifically, the authors estimated gene pleiotropy, approximated by a tissue-specificity index that scales with the number of tissues where each gene is expressed, as well as the age of genes expressed at different stages of gametogenesis. Moreover, the authors assessed evolutionary rates of protein-coding genes, which they split into adaptive and non-adaptive fractions, respectively.

These analyses revealed that both gene pleiotropy and the age of genes decrease abruptly during spermatogenesis from mitotic to meiotic stages (Fig 1A), which agrees with the idea that older genes tend to be more pleiotropic, while younger genes tend to be more tissue-specific. Also, adaptive signals are higher for genes expressed during meiosis, especially in spermatocytes. Non-adaptive signals, in contrast, continue to increase towards the end of spermatogenesis, which suggests a progressive relaxation of coding-sequence constraints over sperm differentiation, a phenomenon commonly observed for other developmental trajectories [8], accompanied by increased expression of evolutionarily young genes.

In the ovary, genes expressed in the germline tend to be pleiotropic and not particularly young, which is in striking contrast to observations in testis. Also, rates of adaptive and non-adaptive coding evolution do not peak in germarium cells (Fig 1B). Interestingly, a few somatic cell types stand out with a slightly higher fraction of younger genes, higher tissue-specificity and higher evolutionary rates of protein-coding genes. Thus, differential gene expression emerges as the only evolutionary characteristic that is clearly elevated in the female germline (Fig 1B).

Of note, genes found to be differentially expressed in the ovary are enriched on the X chromosome, while genes exclusively differentially expressed in the testis (and not the ovary) do not follow this pattern. This asymmetry, together with higher rates of adaptive coding evolution throughout all stages of gametogenesis, and in both sexes (Fig 1), are consistent with a role of female-expressed genes in the faster-X effect. The authors therefore suggest that the faster-X effect in *Drosophila* might be driven by dominant rather than recessive mutations.

Taken together, based on the application of single-nucleus technology in an evolutionary context, Hariyani and colleagues observe distinctive patterns of molecular evolution for genes expressed during spermatogenesis and oogenesis. While their findings for spermatogenesis are in good agreement with earlier observations in mammals [4], their findings for oogenesis are in contrast to recent observations in primates, which reveal elevated molecular evolutionary rates for various metrics also in oogenesis, and a more symmetrical pattern between oogenesis and spermatogenesis [9]. However, it must be noted that their resolution remains lower for oogenesis than for spermatogenesis, which could blur more fine-grained signals in oogenesis and thereby contribute to the observed asymmetry [6]. Thus, even though the study by Hariyani and colleagues is exemplary in its approach, high-resolution comparative analyses of oogenesis across a wider range of taxa are needed to confirm and establish common patterns of molecular evolution.

Another promising research direction following in their footsteps could be a focus on closely related species in an ecological context of speciation research. Importantly, such an application would permit dissecting how selective pressures acting on oogenesis and spermatogenesis build up during speciation and contribute to gene misregulation in interspecific hybrids. A first step in this direction is taken by a recent study in flycatchers [10], where the comparative angle of male and female gametogenesis and the inclusion of hybrids delineate tantalizing future steps.
